# New 1,3-benzodioxin-4-ones from *Synnemapestaloides ericacearum* sp. nov., a biosynthetic link to remarkable compounds within the Xylariales

**DOI:** 10.1371/journal.pone.0198321

**Published:** 2018-06-27

**Authors:** Joey B. Tanney, Justin B. Renaud, J. David Miller, David R. McMullin

**Affiliations:** 1 Department of Biology, Carleton University, Ottawa, Ontario, Canada; 2 London Research and Development Centre, Agriculture and Agri-Food Canada, London, Ontario, Canada; 3 Department of Chemistry, Carleton University, Ottawa, Ontario, Canada; Georg-August-Universitat Gottingen, GERMANY

## Abstract

Surveys of foliar endophytes from the Acadian forest region over the past three decades have identified numerous phylogenetically diverse fungi producing natural products toxic to forest pests and diseases. The life histories of some conifer endophytes can be restricted to plant foliage or may include saprotrophic phases on other plants tissues or even alternate hosts. Considering the potentially broad host preferences of conifer endophytes we explored fungi isolated from understory species and their metabolites as part of an ongoing investigation of fungal biodiversity from the Acadian forest. We report a hitherto unidentified Xylariomycetidae species isolated from symptomatic Labrador tea (*Rhododendron groenlandicum)* leaves and mountain laurel (*Kalmia latifolia*) collected in coastal southern New Brunswick, Canada. Morphological and phylogenetic evidence demonstrated the unknown species was a novel *Synnemapestaloides* (Sporocadaceae) species, described here as *Syn*. *ericacearum*. A preliminary screening assay indicated that the culture filtrate extract of the new species was potently antifungal towards the biotrophic pathogen *Microbotryum violaceum*, warranting an investigation of its natural products. Two natural products possessing a rare 1,3-benzodioxin-4-one scaffold, synnemadoxins A-B (**1–2**), and their postulated precursor, synnemadiacid A (**3**), were characterized as new structures and assessed for antimicrobial activity. All isolated compounds elicited *in vitro* inhibitory antifungal activity towards *M*. *violaceum* at 2.3 μg mL^-1^ and moderate antibiotic activity. Further, the characterization of synnemadoxins A-B provided a perspective on the biosynthesis of some related 1,3-benzodioxin-4-ones produced by other fungi within the Xylariales.

## Introduction

Foliar endophytes of conifers in the Acadian forest of Eastern Canada have proven an exceptionally rich source of structurally diverse biologically active natural products [1,2]. Our studies have revealed that the life histories of conifer endophytes can be restricted to plant foliage or may include saprotrophic phases on other host tissues or even alternate hosts. For example, *Phialocephala scopiformis* is a common *Picea* endophyte that produces its sexual reproductive structures (apothecia) on decaying *Picea* wood and fallen branches [3]. In contrast, apothecia of *Phialocephala piceae*, a foliar endophyte of *Picea*, are reported only from decaying hardwood [3–5]. A griseofulvin-producing *Xylaria* endophyte of *Pinus strobus* was also isolated as an endophyte of *Vaccinium angustifolium*, indicating a plurivorous life cycle involving both overstory and understory species [6].

While considering the potentially broad host preferences of conifer endophytes, an unidentified species in Xylariomycetidae was isolated from symptomatic *Rhododendron groenlandicum* leaves. The resulting strain, tentatively identified as a *Seimatosporium* species, generated a potently antifungal culture filtrate extract, prompting further investigation. Morphological and phylogenetic analyses indicated this strain, as well as a conspecific strain isolated from symptomatic *Kalmia latifolia* leaves, represented a novel *Synnemapestaloides* (Sporocadaceae) species. Herein, we describe the new species as *Synnemapestaloides ericacearum*, providing morphological and phylogenetic evidence supporting its distinction from *Synnemapestaloides* and other morphologically similar species within the Sporocadaceae. From the culture filtrate extract of *Syn*. *ericacearum* DAOMC 250336, three new natural products, including two metabolites possessing a rare 1,3-benzodioxin-4-one scaffold, were structurally characterized and assessed for antimicrobial activity.

## Results

**Fig 1:**
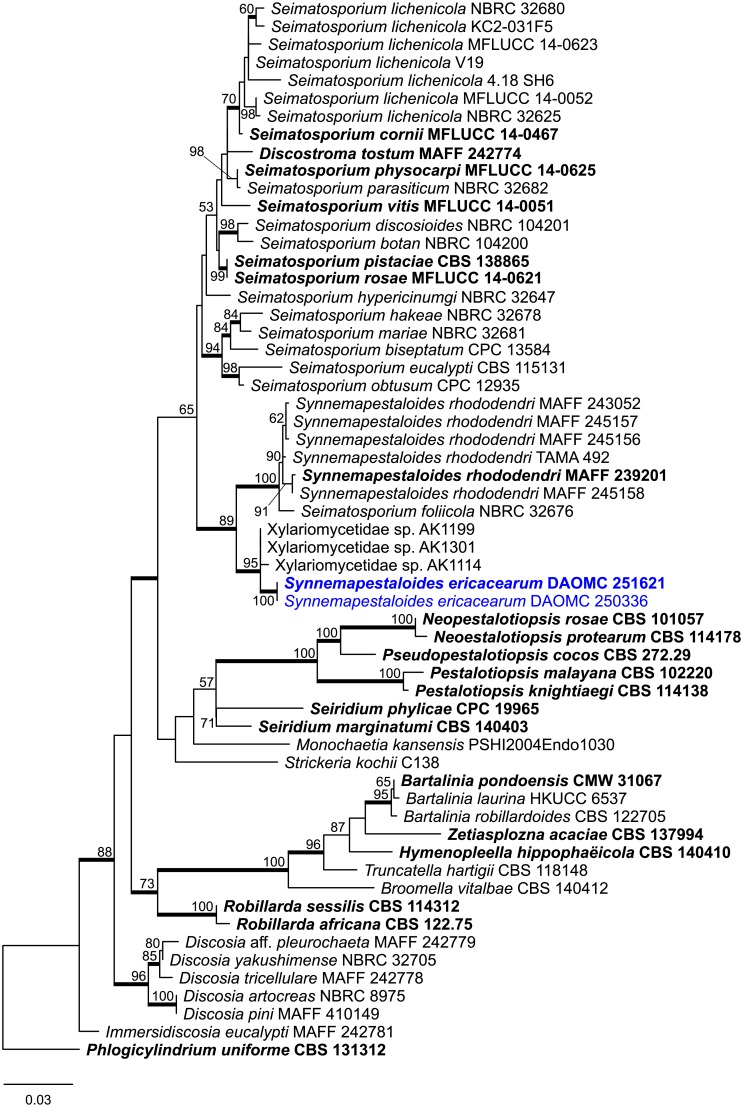
Most likely tree from a RAxML analysis of ITS-LSU dataset containing representative Sporocadaceae species. Culture collection accession numbers or specimen identifiers follow the species name, with sequences from type specimens or ex-type strains in bold. RAxML bootstrap support percentages ≥50 from a summary of 1000 replicates are presented at the branch nodes. Thickened branches indicate Bayesian posterior probability values ≥0.95. The tree was rooted with *Phlogicylindrium uniforme* CBS 131312 and the scale bar represents the number of substitutions per site.

Strains from symptomatic leaves of *Rhododendron groenlandicum* (collected on Taylors Island, Saint John, NB, 45.212018, -66.136745) and *Kalmia latifolia* (see below) were conspecific based on morphology and identical ITS and LSU sequences. Both isolates were sterile on all media tested; however, sporulation by one strain (DAOMC 250336) was induced when malt extract agar (MEA) blocks containing mycelia were floated in sterile water. This species is morphologically similar to *Seimatosporium* (Sporocadaceae), a plurivorous genus including several species described from hosts in the Ericaceae, for example *Seim*. *arbuti*, *Seim*. *ledi*, and *Seim*. *rhododendri* on *Rhododendron* [7,8]. The ITS-LSU phylogeny places both strains in a strongly supported monophyletic clade sister to *Synnemapestaloides rhododendri* and *Syn*. *foliicola* (Fig 1). Based on morphological and phylogenetic evidence, we consider these two strains to represent a new species, described herewith as *Synnemapestaloides ericacearum*.

Based on a NCBI GenBank BLAST query using *Syn*. *ericacearum* ITS sequences, three unidentified endophyte sequences (Xylariomycetidae sp.) were included in the ITS-LSU phylogeny. These endophyte sequences are of strains isolated from surface-sterilized photosynthetic tissues of *Equisetum arvense* (JQ759638, JQ759542) and the Ericaceae host *Cassiope tetragona* (JQ759462) in Alaska, U.S.A. [9]. The unidentified endophyte ITS sequences were very similar to, and probably conspecific with, *Syn*. *ericacearum*, e.g.: JQ759638: identities = 734/738 (99%), gaps = 1/738 (0%).

### Taxonomy

**Fig 2:**
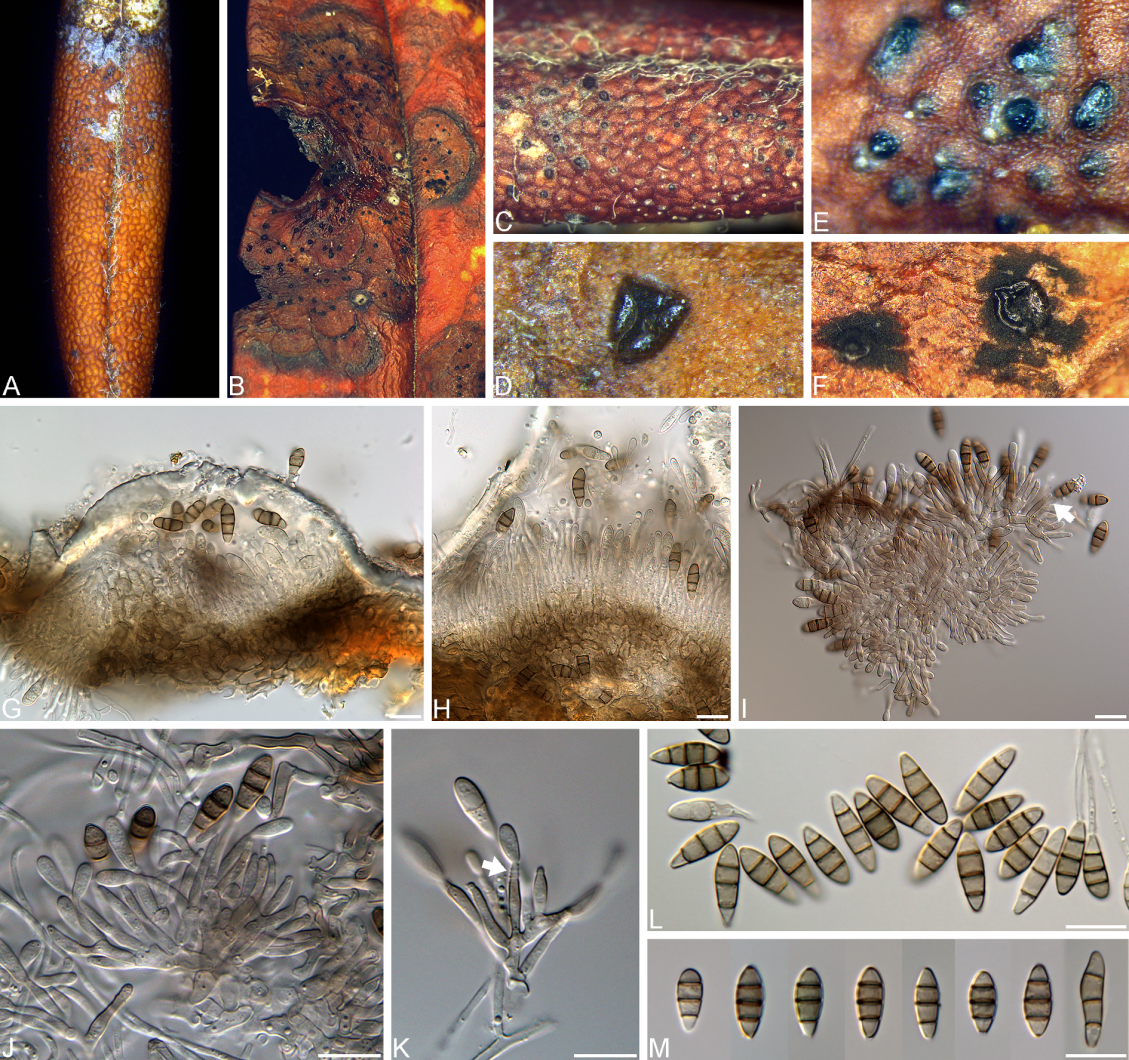
*Synnemapestaloides ericacearum*. A, C, E. Leaf spots and acervuli on *Rhododendron groenlandicum* (DAOM 745782). B, D, F. Coalescing leaf spot and acervuli on *Kalmia latifolia* (DAOM 867445, holotype), triangular acervuli in F. G. Acervulus with intact host cuticle. H. Acervulus with ruptured host cuticle. I–K. Conidiophores and conidia, arrows denoting conidiogenous cells exhibiting obvious successive percurrent extensions. L–M. Conidia. Scale bars: G–M = 10 μm.

*Synnemapestaloides ericacearum* J.B. Tanney, sp. nov. (Figs 1 and 2)

MycoBank MB 824126

Typification. CANADA. NEW BRUNSWICK: Charlotte County, Roosevelt Campobello International Park, near Upper Duck Pond, 44.849370, -66.965173, from symptomatic *Kalmia latifolia* leaves, 26 Sep 2016, *J*.*B*. *Tanney NB-846* (**holotype** DAOM 867445). Ex-type culture DAOMC 251621.

Etymology: Named for its association with Ericaceae hosts.

ITS barcode: MG687266 (*LSU* = MG687267)

Leaf spots mostly epiphyllous, sometimes hypophyllous, punctiform to effused, circular to lobate, discrete, eventually becoming irregularly confluent and covering most of the leaf lamina, distinct on upper surface but less conspicuous on lower surface, brown, delimited from healthy leaf tissue by thin purple-brown margin.

Colonies 40–45 mm diam after 7 d in 12:12 h light/dark at 20 °C on MEA; flat, sparse to moderately abundant hyaline aerial mycelia, margin entire, flat, wide, hyaline; surface and reverse white to reddish blond (5C4) in center. Exudates and soluble pigments absent. Mycelium consisting of hyaline, smooth, septate, branched, hyphae 2–8 μm diam.

Conidiomata acervular, pulvinate, circular or triangular in outline, up to 275 μm diam, blackish-brown to black, glabrous, epiphyllous, subcuticular, scattered, initially covered by the host cuticle, which becomes raised then irregularly ruptures laterally or radially to release black mass of conidia; basal stroma well-developed, up to 30 μm thick, pale brown *textura angularis* composed of thick-walled, pale brown cells (3–)4–6(–7.5) μm diam, inner wall becoming hyaline with thinner-walled cells.

Conidiophores aggregated into a compact layer, up to 30–55 μm long, 2–3(–5.5) μm wide, septate, branched, hyaline, smooth, thin-walled. Conidiogenous cells holoblastic, annellidic, cylindrical to slightly ampuliform, hyaline, smooth, thin-walled, bearing up to six successive percurrent extensions, (7–)8.5–11.5(–12.5) × 2–2.5(–3) μm (length: n = 30, $\bar{\text{x}}=9.8$ μm, SD = 1.5 μm, SE = 0.29 μm, 95% CI = 0.57; width: n = 30, $\bar{\text{x}}=2.0$ μm, SD = 0.3 μm, SE = 0.05 μm, 95% CI = 0.1).

Conidia fusiform-ellipsoid to fusiform-cylindrical or clavate, smooth, 3-septate, pale brown to brown, concolorous when young, becoming versicolorous with age, (10.5–)11.5–13.5(–16.5) × (3.5–)4–5(–5.5) μm (length: n = 73, $\bar{\text{x}}=12.5$ μm, SD = 1 μm, SE = 0.12 μm, 95% CI = 0.24; width: n = 73, $\bar{\text{x}}=4.5$ μm, SD = 0.36 μm, SE = 0.04 μm, 95% CI = 0.08); apical cell elongate-conic, sometimes apiculate, usually same wall-thickness but sometimes paler than median cells, (2–)3–4(–4.5) μm long (n = 55, $\bar{\text{x}}=3.5$ μm, SD = 0.5 μm, SE = 0.07 μm, 95% CI = 0.14); two median cells doliiform to subcylindrical, moderately thick-walled, pale brown to brown; penultimate cell 2.5–3.5(–4) μm long (n = 55, $\bar{\text{x}}=3$ μm, SD = 0.5 μm, SE = 0.06 μm, 95% CI = 0.13); antepenultimate cell (2.5–)3–3.5(–4) μm long (n = 55, $\bar{\text{x}}=3$ μm, SE = 0.4 μm, SE = 0.05 μm, 95% CI = 0.10); basal cell obconic, thin-walled, subhyaline or occasionally hyaline, subtruncate to truncate with unthickened, 1–1.5 μm diam basal scar that is sometimes inconspicuous, (2–)2.5–3.5(–4) μm long (n = 55, $\bar{\text{x}}=3$ μm, SD = 0.4 μm, SE = 0.06 μm, 95% CI = 0.12); appendages absent.

Host range: Causing leaf spots on *Kalmia latifolia* and *Rhododendron groenlandicum*.

Distribution: Canada (New Brunswick).

Additional specimens and cultures examined: CANADA. NEW BRUNSWICK: Saint John County, Saint John, Taylors Island, 45.212018, -66.136745, from symptomatic *Rhododendron groenlandicum* leaves, 26 Sep 2016, *A*.*K*. *Walker DAOM 745782/DAOMC 250336*, ITS barcode: MG687268 (LSU = MG687269).

Based on ITS-LSU phylogeny, *Synnemapestaloides ericacearum* is sister to a clade containing *Syn*. *foliicola* and *Syn*. *rhododendri*. *Synnemapestaloides rhododendri* produces synnemata that give rise to five-septate conidia with appendages [10], while *Syn*. *ericacearum* produces three-septate conidia lacking appendages from acervuli. *Synnemapestaloides foliicola* differs from *Syn*. *ericacearum* by its five-septate conidia bearing terminal appendages produced from sporodochia with similar ontogeny to that of *Syn*. *rhododendri* [10,11]. The absence of conidial appendages distinguishes *Syn*. *ericacearum* from some other *Seimatosporium* species reported from Ericaceae hosts, e.g.: *Seim*. *arbuti* and *Seim*. *azalea* conidia possess terminal appendages [12,13] and *Seim*. *ledi* conidia possess basal appendages [7]. *Seimatosporium rhododendri* conidia are three-septate and unappendaged, but significantly larger than those of *Syn*. *ericacearum* (15.5–20 × 6.5–8.5 μm) [7]. Similarly, the conidia of *Seim*. *vaccinii* are three-septate and unappendaged, but are somewhat longer (13–18 × 4.5–5.5 μm) than those of *Syn*. *ericaearum*, and also born from shorter conidiophores (up to 28 μm long vs. 30–55 μm long) [14]. The mostly three-septate, unappendaged conidia of *Seim*. *lichenicola* are similar to those of *Syn*. *Ericacearum*; however, they are considerably larger: 18–20 × 5–7 um versus (10.5–)11.5–13.5(–16.5) × (3.5–)4–5(–5.5) μm [15], although Sutton [14] reported *Seim*. *lichenicola* conidial dimensions more similar to those of *Syn*. *ericacearum*: 13–15 × 4.4–6.5 μm. Norphanphoun et al. [16] designated a reference specimen of *Seim*. *lichenicola* from *Cotinus coggygria* with conidial dimensions similar to *Syn*. *ericacearum* and *Seim*. *lichenicola* sensu Sutton [14], (10–)12–14 × 4–5(–6) μm; however, it is phylogenetically distinct.

### Natural products

**Fig 3:**
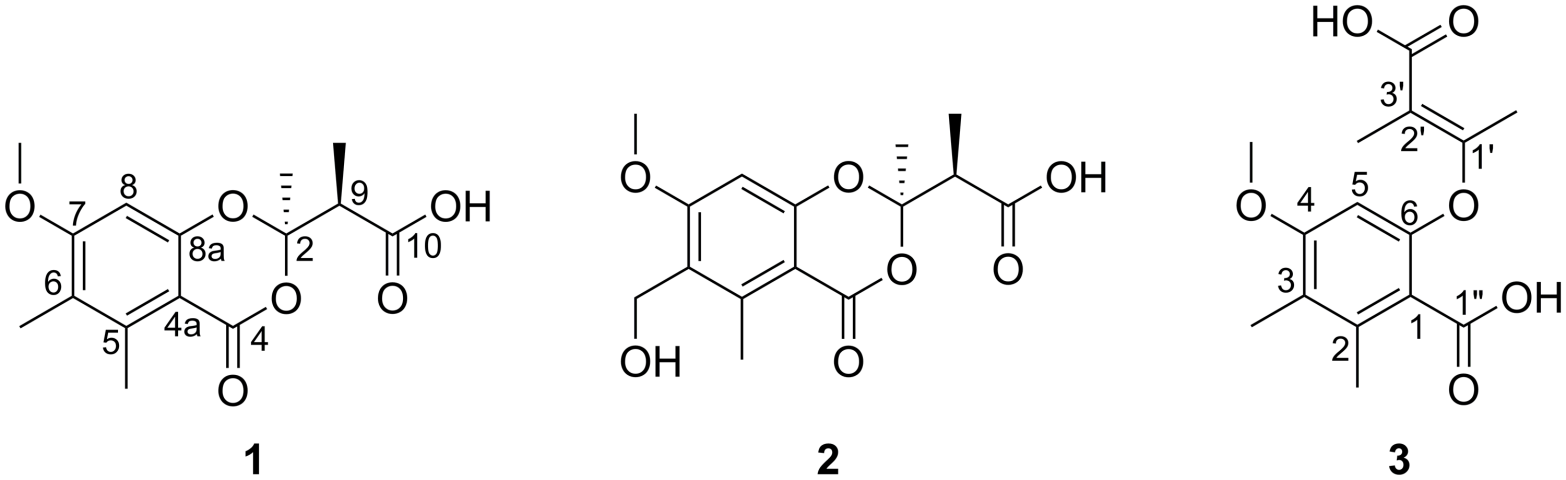
Structures of new 1,3-benzodioxin-4-ones synnemadoxins A-B (1–2) and synnemadiacid A (3) characterized from *Syn*. *ericacearum* DAOMC 250336.

**Fig 4:**
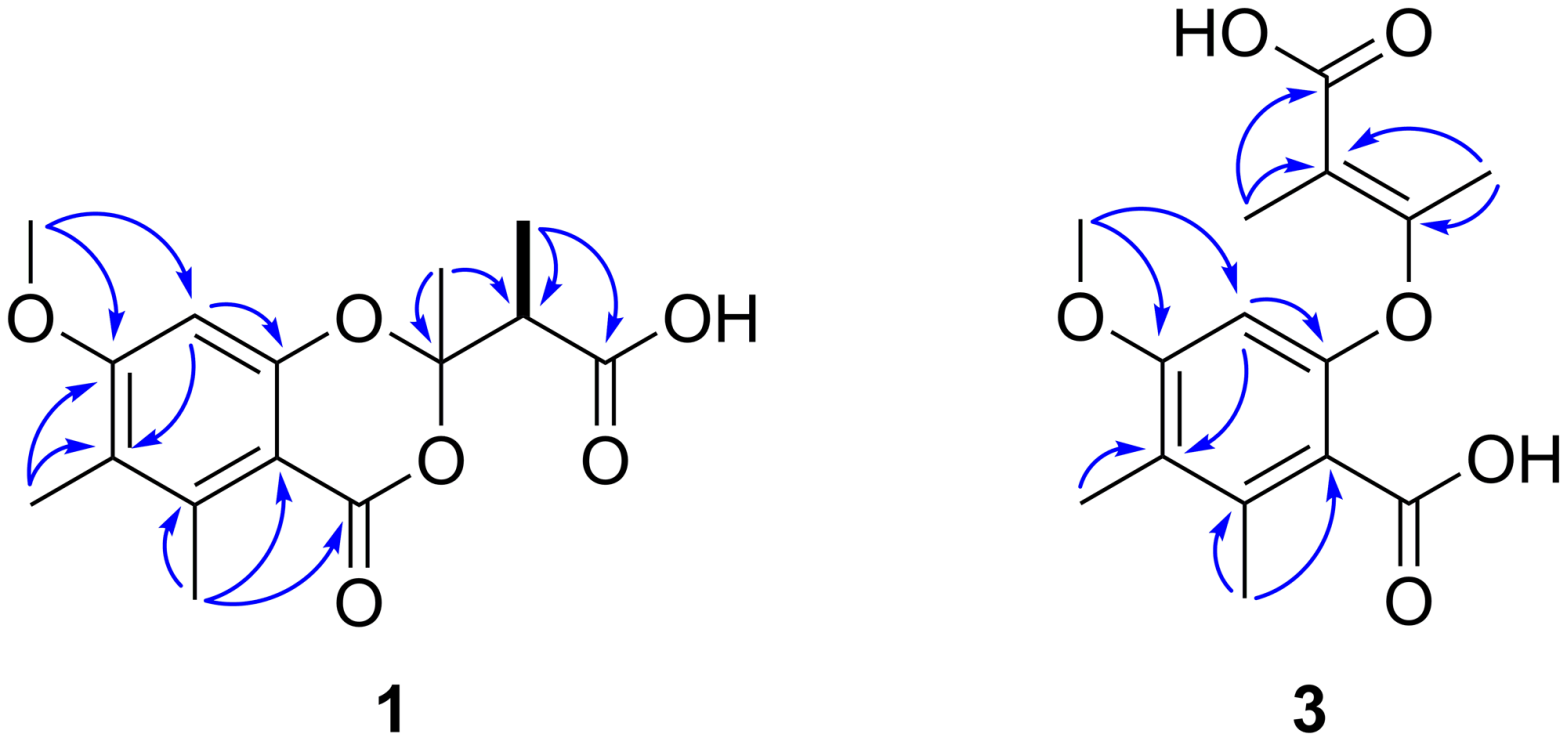
Observed COSY (bold) and key HMBC (blue) correlations for synnemadoxin A (1) and synnemadiacid A (3).

The incubation of *Syn*. *ericacearum* DAOMC 250336 in 2% malt extract broth yielded ethyl acetate culture filtrate extracts that exhibited strong *in vitro* antifungal activity using a modified Oxford diffusion assay. Three new natural products including two rare 1,3-benzodioxin-4-ones were structurally characterized and assessed for antimicrobial activity (Fig 3). The first metabolite (**1**) purified was isolated as a dull brown oil with the molecular formula C_15_H_18_O_6_ determined by an [M-H]—ion at *m/z* 293.1033 indicative of seven units of unsaturation. Its UV spectrum showed absorption maxima at 224, 268, and 306 nm suggesting the presence of a conjugated structure. Its ^1^H NMR spectrum displayed a single aromatic methine at *δ* 6.51 (s), deshielded methine at *δ* 3.18 (q, 7.0), and five methyl groups at *δ* 2.58 (s), 2.11 (s), 1.73 (s), and 1.28 (d, 7.0) as well as a methoxy group at *δ* 3.87 (s). Interpretation of the HSQC and ^13^C NMR spectra of **1** revealed eight of the fifteen carbon signals were quaternary. These signals were resultant of two carbonyls at *δ* 175.4 and 161.9, five sp^2^ carbons at *δ* 165.0, 157.7, 143.2, 122.7, and 106.2 and one sp^3^ carbon at *δ* 107.2 (Table 1). The presence of eight highly deshielded quaternary carbons and four singlet methyl groups supported a highly substituted, conjugated structure. As expected from the ^1^H spectrum, only one COSY cross-peak was observed for compound **1**, between *δ* 1.28 (9-Me) and *δ* 3.18 (H-9; Fig 4). HMBC correlations from the singlet methyl at *δ* 1.73 (2-Me) to *δ* 107.2 (C-2) and 47.8 (C-9), the cross peak from 9-Me to the carbonyl at *δ* 175.4 (C-10) and the chemical shift of C-2 suggested the presence of a 3,3-dioxy-2-methylbutanoic acid moiety in **1** (Fig 4). The presence of a single carboxylic acid (C-10) was supported by methylation of **1** with excess diazomethane that yielded a hexamethyl derivative (**1a**), identifying one exchangeable acidic proton within the structure (Figure J and Table E in S1 File). The high number of deshielded quaternary carbons and extensive HMBC coupling for **1** suggested a pentasubstituted aromatic ring. A lactone or other ring system fused to the aromatic moiety possessing a carbonyl at *δ* 161.9 (C-4) was theorized to constitute the remaining degrees of unsaturation. HMBC correlations from the singlet methyl at *δ* 2.11 (6-Me) to *δ* 143.2 (C-5), 122.7 (C-6) and (C-7), and from *δ* 3.87 (7-OMe) to *δ* 165.0 (C-7) and 98.1 (C-8) revealed a methoxy functionality vicinal to the only aromatic methine proton. HMBC cross-peaks were also observed from *δ* 2.58 (5-Me) to *δ* 161.9 (C-4), 106.2 (C-4a) and C-5, and from *δ* 6.51 (H-8) to C-4a, C-6, C-7 and C-8a (*δ* 157.7; Fig 4). These data and the chemical shift of C-8a revealed that compound **1** possess a modified 6-hydroxy-4-methoxy-2,3-dimethylbenzoic acid core structure. Together, the chemical shift of C-2, lack of other exchangeable acidic protons and an HMBC correlation from 2-Me to C-4 fuses the previously characterized 3,3-dioxy-2-methylbutanoic acid moiety to the aromatic functionality yielding a rare 1,3-benzodioxin-4-one structure (Figs 3 and 4). The 1,3-dioxin-4-one ring satisfies the seventh unit of unsaturation dictated by the molecular formula and NMR data.

**Table 1:** ^1^H (400 MHz) and ^13^C (100 MHz) NMR data for synnemadoxins A-B (1–2) in CD_3_OD.

Position	1		2	
	*δ*_C_, type	*δ*_H_ (*J* in Hz)	*δ*_C_, type	*δ*_H_ (*J* in Hz)
**2**	107.2, C		107.3, C	
**4**	161.9, C		161.5, C	
**4a**	106.2, C		106.4, C	
**5**	143.2, C		145.7, C	
**6**	122.7, C		125.4, C	
**7**	165.0, C		165.6, C	
**8**	98.1, CH	6.51, s	98.6, CH	6.57, s
**8a**	157.7, C		159.3, C	
**9**	47.8, CH	3.18, q (7.0)	47.8, CH	3.19, q (7.0)
**10**	175.4, C		175.0, C	
**2-Me**	20.9, CH_3_	1.73, s	21.0, CH_3_	1.74, s
**5-Me**	17.4, CH_3_	2.58, s	17.0, CH_3_	2.71, s
**6-Me**	11.3, CH_3_	2.11, s	55.0, CH_2_	4.70, s
**9-Me**	12.9, CH_3_	1.28, d (7.0)	12.9, CH_3_	1.29, d (7.0)
**7-OMe**	56.6, CH_3_	3.87, s	56.7, CH_3_	3.90, s
**10-OMe**				

The planar 1,3-benzodioxin-4-one structure of **1** was elucidated as a new natural product; however, the configurations of the adjacent chiral centers at C-2 and C-9 remained elusive. Due to the lability of the 3,3-dioxy-2-methylbutanoic acid moiety, conventional NOESY experiments used to resolve stereochemical assignments were not discriminatory. Attempts to generate crystals of compound **1** or its potassium salt were also not successful. Chiral HPLC of **1** revealed an approximate 55:45 mixture of enantiomers, dominated by the positive enantiomer according to the optical rotation values, [α]^23^_D_ 9.4 (c 0.3, MeOH), [α]^23^_D_ 9.8 (c 0.3, CHCl_3_). To determine the relative configurations of C-2 and C-9, the *in silico* approach described by Willoughby et al. 2017 [17] was used. Briefly, molecular dynamics were calculated with AMBER (University of California) to generate a library of all possible conformers for each stereochemical configuration. Density function theory was used to calculate frequency and free energy values for each conformation to generate *in silico* computed chemical shifts. Comparisons of mean absolute error (MAE) between experimental and computed chemical shifts of all reported ^1^H and ^13^C resonances for **1** and its methyl ester **1a** revealed the relative configurations of the enantiomeric mixture of **1** as 2*R*,9*R* and 2*S*,9*S* (Tables A-D in S1 File). This *in silico* approach is not able to differentiate between enantiomers of **1**; however, is discriminatory of diastereomers. The HRMS and spectroscopic data support **1** as a new rare 1,3-benzodioxin-4-one structure reported here as synnemadoxin A (Fig 3).

The second metabolite characterized from *Syn*. *ericacearum* DAOMC 250336 was also isolated as dull brown oil with the same UV absorption maxima as synnemadoxin A (**1**). Metabolite **2** was more polar and assigned the molecular formula C_15_H_18_O_7_ based on an HRMS peak at *m/z* 309.0981 [M-H]^-^ corresponding to the addition of an oxygen atom compared to synnemadoxin A (**1**). The NMR spectroscopic data for **2** were very similar to synnemadoxin A (**1**), indicating **2** was a structurally similar 1,3-benzodioxin-4-one metabolite (Table 1). The primary difference between the two 1,3-benzodioxin-4-ones NMR data was the substitution of a methyl group in synnemadoxin A (**1**) with an oxygenated methylene in **2** revealed by ^1^H and ^13^C signals at *δ* 4.70 (s) and *δ* 55.0, respectively (Table 1). Together, the differences in NMR chemical shifts, increase in molecular weight, and HMBC correlations from *δ* 4.70 (6-CH_2_OH) to *δ* 145.7 (C-5), 125.4 (C-6) and 165.6 (C-7), are indicative of C-6 bearing a hydroxymethyl moiety in **2** instead of a methyl group (Fig 3). The two 1,3-benzodioxin-4-ones (**1–2**) similar ^1^H NMR chemical shift data, and positive optical rotation values, [α]^23^_D_ 10.4 (c 0.3, MeOH) for **2**, are evidence for the adjacent chiral centers having the same configurations as synnemadoxin A (**1**). Here we report **2** as a new structure, synnemadoxin B.

Compound **3** was purified as the major constituent of the *Syn*. *ericacearum* DAOMC 250336 culture filtrate extract as a yellow oil with the same molecular formula as synnemadoxin A (**1**) determined by an HRMS peak at *m/z* 293.1032 [M-H]^-^. Based on resonances in the ^1^H and ^13^C NMR spectra, compound **3** possesses a pentasubstituted aromatic ring similar to synnemadoxin A (**1**) where primary differences in the NMR spectra of synnemadoxin A (**1**) and **3** appeared in the acyclic moiety (Tables 1 and 2). Chemical shifts for **3** at *δ* 162.8 (C-1’) and 113.0 (C-2’), the absence of an NOESY correlation between vicinal methyl protons at *δ* 2.20 (1’-Me) and 1.90 (2’-Me), and magnitude of their coupling constant (*J* = 1.4 Hz) reveals vicinal methyl protons *trans-* configuration on a tetrasubstituted double bond (Fig 3). An additional carbonyl carboxylic acid chemical shift at *δ* 171.8 (C-1”) and *trans*-double bond together with the HRMS data, and similar NMR spectra suggested a reduction of the 1,3-dioxin-4-one ring in **3** (Fig 4). Here were report compound **3** as a new structure, (*E*)-6-((3-carboxybut-2-en-2-yl)oxy)-4-methoxy-2,3-dimethylbenzoic acid, or synnemadiacid A (Fig 3).

**Table 2:** ^1^H (400 MHz) and ^13^C (100 MHz) NMR data for synnemadiacid A (3) CD_3_OD.

Position	3	
	*δ*_C_, type	*δ*_H_ (*J* in Hz)
**1**	122.0, C	
**2**	136.4, C	
**3**	122.2, C	
**4**	160.0, C	
**5**	100.8, CH	6.36, s
**6**	151.0, C	
**2-Me**	17.1, CH_3_	2.26, s
**3-Me**	11.5, CH_3_	2.11, s
**1’**	162.8, C	
**2’**	113.0, C	
**3’**	172.4, C	
**1’-Me**	17.5, CH_3_	2.20, q (1.4)
**2’-Me**	12.6, CH_3_	1.90, q (1.4)
**1”**	171.8, C	
**4-OMe**	56.2, CH_3_	3.79, s
**3’-OMe**		
**1”-OMe**		

Natural products **1–3** were individually tested for *in vitro* antifungal activity against *Microbotryum violaceum* and *Saccharomyces cerevisiae*, and antibiotic activity towards *Bacillus subtilis* and *Escherichia coli* (Table 3). Synnemadoxins A-B (**1**–**2**) and synnemadiacid A (**3**) were potently inhibitory to *M*. *violaceum* (formerly *Ustilago violacea*), a proxy for rust needle diseases [18], each with a MIC of 2.3 μg mL^-1^. However, no activity was observed for each metabolite at the highest concentration tested (150 μg mL^-1^) against *S*. *cerevisiae* despite the crude culture filtrate extract showing activity at 50 mg mL^-1^ using a modified Oxford diffusion assay. Moderate antibiotic activity for compounds **1–3** against *B*. *subtilis* were observed at 9.3 μg mL^-1^; and **1** and **3** had MIC values of 18.8 μg mL^-1^ against *E*. *coli*.

**Table 3:** Minimum inhibitory concentrations for 1–3 isolated from *Syn*. *ericacearum* DAOMC 250336.

compound	MIC (μg mL^-1^)			
	*M*. *violaceum*	*S*. *cerevisiae*	*B*. *subtilis*	*E*. *coli*
**1**	2.3	-	9.3	18.8
**2**	2.3	-	9.3	-
**3**	2.3	-	9.3	18.8

- No inhibitory observed at highest concentration tested (150 μg mL^-1^)

## Discussion

*Synnemapestaloides* was established to describe *Syn*. *rhododendri*, the causal agent of a leaf and twig blight disease of *Rhododendron brachycarpum* in Japan [10]. Handa et al. [10] provided morphological evidence distinguishing the monotypic *Synnemapestaloides* from other pestaloid genera, namely the presence of columnar synnemata. A later phylogenetic study confirmed the distinction of *Synnemapestaloides* from *Seimatosporium* and other Sporocadaceae genera, while also establishing the new combination *Syn*. *foliicola* for a presumably saprotrophic species known from *Chamaecyparis* and *Juniperus*, previously placed in *Seimatosporium* [19,20]. The unappendaged three-septate conidia of *Synnemapestaloides ericacearum* distinguish it from the five-septate conidia of both *Syn*. *foliicola* and *Syn*. *rhododendri*. *Synnemapestaloides rhododendri* conidia are fusiform and more-or-less pestaloid, with unbranched or dichotomously branched apical appendages and unbranched or irregularly branched basal appendages, while *Syn*. *foliicola* conidia are broadly ovate with unbranched apical and basal appendages [10,20]. Watanabe et al. [19] described similar conidiomata ontogeny for both *Syn*. *rhododendri*, which produces up to ca. 500 μm tall synnemata, and *Syn*. *foliicola*, which produces short synnemata (sporodochia). The authors hypothesized that this conidiomata ontogeny might be a synapomorphy for *Synnemapestaloides*. We interpret the conidiomata of *Syn*. *ericacearum* as acervular, with the conidiogenous cells and conidia observed under the ruptured leaf cuticle (Fig 2G and 2H). However, detailed time-lapse observations would be necessary to elucidate the conidiomata ontogeny of *Syn*. *ericacearum*. There are no reports of sexual states in *Synnemapestaloides*.

Currently, *Syn*. *ericacearum* is only known as an associate of leaf spots on the Ericaceae hosts *Kalmia latifolia* and *Rhododendron groenlandicum*. However, it is conceivable that the life cycle of *Syn*. *ericacearum* contains an endophytic phase, which is common for species from the Sporocadaceae (e.g., [13, 21, 22]). The association of *Syn*. *ericacearum* with leaf spots characteristic of those caused by *Syn*. *rhododendri*, *Seimatosporium*, and other Sporocadaceae species indicate it is likely a pathogen, although its pathogenicity was not tested.

Many strains of fungi within Sporocadaceae representing the genera *Pestalotiopsis*, *Bartalinia*, *Monochaetia*, *Robillarda*, *Seimatosporium*, and *Strickeria* have been critically evaluated for natural products [23]. This interest resulted from the putative identification of the plant anticancer compound paclitaxel (taxol) from *Taxomyces andreanae*, collected as an endophyte of *Taxus brevifolia*, and subsequently from several related species including endophytic strains of *Bartalinia*, *Monochaetia*, and *Pestalotiopsis* [24]. Horizontal transfer of biosynthetic genes from plants to fungi was postulated to explain paclitaxel production by these fungi. However, more recent evidence shows the genomes of the putative endophyte producers’ do not possess any significant homology to plant paclitaxel biosynthetic genes, questioning its biosynthesis by these fungi [25].

Strains of species within Sporocadaceae collected as endophytic or plant-associates have proved to be an abundant source of structurally diverse natural products. The polyhydroylated macrolides seimatopolides A and B with peroxisome proliferative activity were characterized from a *Seimatosporium discosioides* (GenBank: EF600969) strain isolated from *Rosa multiflora* [26,27]. A *Seimatosporium* endophyte of *Salsola oppositifolia* synthesized several aromatic compounds acid together with metabolites structurally similar to terreic acid, an antiinsectan metabolite isolated from a *Picea* endophyte [1,28]. From the same plant host, the acaranoic acids, seimatoporic acid A-B were reported together with (*R*)-mellein and five of its derivatives [29]. The simple isocoumarin (*R*)-mellein and its derivatives have been reported from phylogenetically diverse plant-associated fungi including *Pezicula*, *Diaporthe* and Rhytismatales species [30–34]. *Pestalotiopsis*, *Robillarda* and other related but unidentified species likely associated with woody plants (collected from three-toed sloth hair) generated cytotoxic and antibiotic culture filtrate extracts; however, metabolite structures were not elucidated [35].

**Fig 5:**
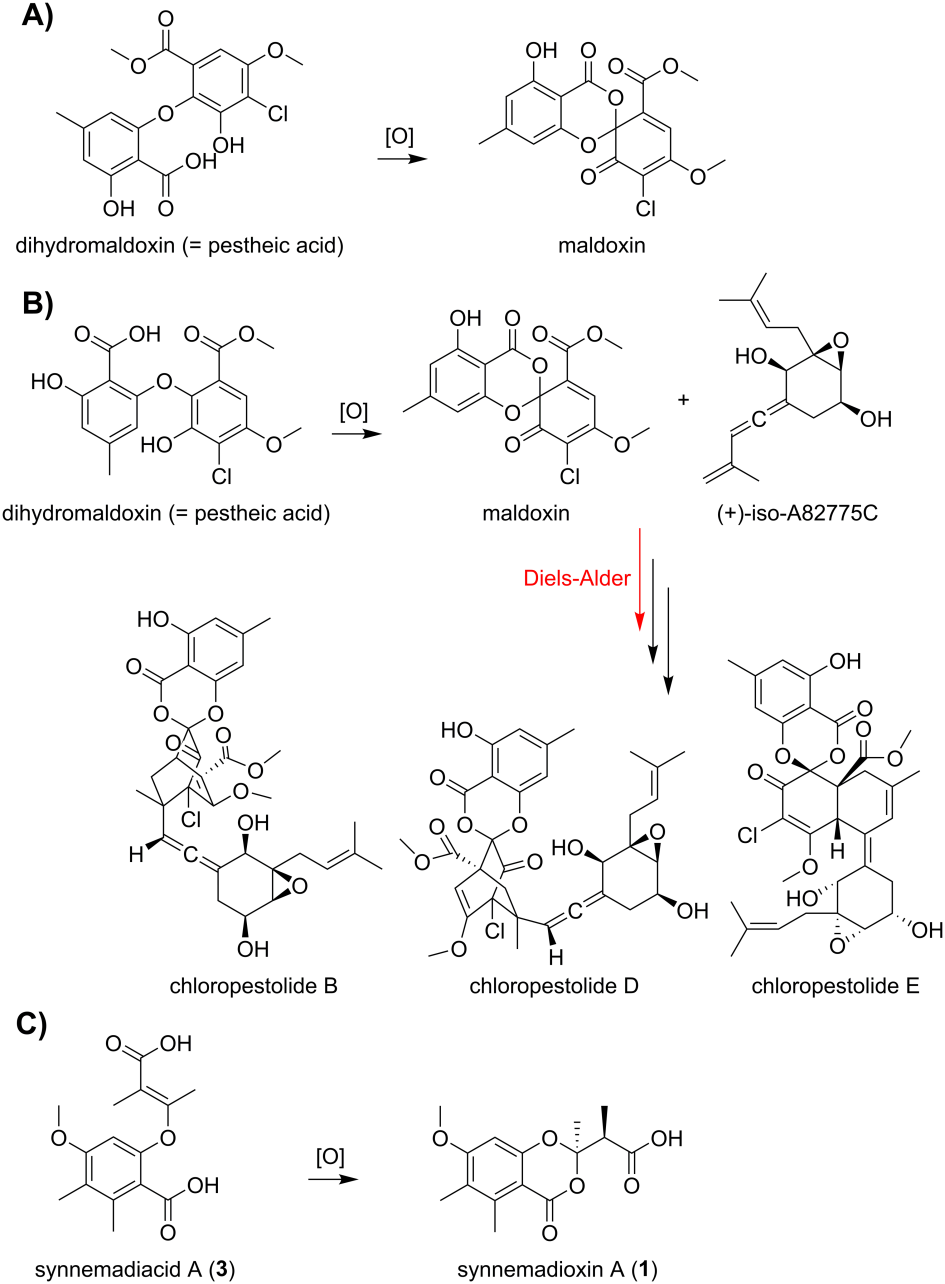
**A**) Proposed biosynthesis of maladoxin from the oxidation of dihydromaldoxin by a Xylariales fungus (unidentified *Xylaria* sp.; adapted from Adeboya et al. 1996). **B**) Proposed biosynthetic scheme of chloropestolides from *P*. *fici* from the oxidation of the diphenyl ether pestheic acid to maladoxin followed by the Diels-Alder addition of iso-A82775C (adapted from Liu et al. 2013). **C**) Proposed biosynthesis of synnemadoxin A (**1**) by *Syn*. *ericacearum* from the oxidation of synnemadiacid A (**3**), analogous to the schemes proposed for maldoxin from and unidentified *Xylaria* sp. (Xylariales) and *P*. *fici*.

Fungal natural products possessing a 1,3-benzodioxin-4-one scaffold present within synnemadoxins A-B (**1–2**) are uncommon. An unidentified *Xylaria* isolate collected from the Malaysian rain forest produced the chlorinated spirocyclohexadienone maladoxin which contains a 1,3-benzodioxin-4-one moiety together with the related chlorinated depsidone maldoxone and diphenyl ether biosynthetic intermediates dihydromaldoxin (= pestheic acid), isodihydromaldoxin, and dechlorodihydromaldoxin (Fig 5A) [36]. A *Pestalotiopsis fici* (Sporocadaceae) strain isolated as an endophyte of *Camellia sinensis* (Ericales) related to *Syn*. *ericacearum* biosynthesizes the most compounds reported to date containing this uncommon structural element (Fig 5) [37]. Genomic data for this strain of *P*. *fici* (GenBank: ARNU00000000) strain revealed a high number of extracellular pectinases. Pectin is a major carbohydrate between plants cells suggesting adaptation to its endophytic state [37]. *P*. *fici* metabolites possessing the 1,3-benzodioxin-4-one core structure include chloropestolides A-G [38,39], chloropupukeanolides A-E [40,41], and dechloromaldoxin [39]. Much of the natural product diversity from this strain (> 80 metabolites) is thought to arise from the stereo-unspecific intermolecular Diels-Alder cycloaddition of intermediates followed by carbonyl-ene reactions [37,39,41]. Proposed intermediates include the same biphenyl ether, dihydromaldoxin (= pestheic acid), and the sesquiterpene (+)-iso-A82775C, isolated together with larger 1,3-benzodioxin-4-one containing metabolites (Fig 5B) [39,41]. The disruption of epigenetic regulators in this *P*. *fici* strain resulted in the characterization of additional new polyketides including eleven new macrolides and metabolites sharing structural features of the proposed precursors noted above [42]. Due to the unusual, highly functionalized structures and reported biological activities of *P*. *fici* metabolites possessing a 1,3-benzodioxin-4-one scaffold, their biogenesis has been critically evaluated [39,41,43,44]. Similar Diels-Alder biosynthetic schemes have also been reported for sorbicillin-derived metabolites from the *Trichoderma* section *Longibrachiatum* [45–47].

The biogenesis of these highly functionalized *P*. *fici* metabolites are proposed to originate with the oxidation of dihydromaldoxin (= pestheic acid) to maladoxin, which possesses a reactive diene functionality. Diels-Alder cascades from the reaction of the maladoxin diene and terminal alkene of (+)-iso-A82775C generate the observed structural diversity for this strain [37]. Due to the reactivity of the maladoxin diene moiety, the Diels-Alder reaction in the presence of the terminal diene of (+)-iso-A82775C (Fig 5B) is speculated to occur almost spontaneously, without major enzymatic involvement. Maladoxin was not isolated from rice culture extracts of *P*. *fici*, whereas the less reactive dechloro congener was [41]. The recovery of maladoxin from the mycelium of the previously noted Xylariales (unidentified *Xylaria* sp.) species together with dihydromaldoxin (= pestheic acid) and other related diphenyl ether intermediates suggests a similar biosynthetic pathway, absent the presence of a reactive diene moiety or additional enzymatic input [36]. Since 1,3-benzodioxin-4-one containing natural products are rarely reported, we speculate synnemadoxins A-B (**1**–**2**) arise from the oxidation of synnemadiacid A (**3**) similar to the oxidation of diphenyl ether intermediates to maladoxin (Fig 5A–5C) by related fungi within the Xylariales [36,39,41]. Oxidative cyclization of the diphenyl ether dihydromaldoxin is reported to yield enantiomeric mixtures of the 1,3-benzodioxin-4-ones maldoxin and dechloromaldoxin [39,48]. A similar oxidative scheme using synnemadiacid A (**3**) as a precursor would help explain the observation of enantiomeric synnemadoxins A-B (**1**–**2**).

In summary, we have described a new species, *Syn*. *ericacearum* (Sporocadaceae) from the ericaceous hosts *R*. *groenlandicum* and *Kalmia latifolia*. The culture filtrate extract from *Syn*. *ericacearum* DAOMC 250336 was potently antifungal. Two natural products possessing a rare 1,3-benzodioxin-4-one scaffold, synnemadoxin A-B (**1–2**), and their postulated precursor, synnemadiacid A (**3**), were characterized as new structures and assessed for biological activity. All isolated compounds elicited *in vitro* inhibitory antifungal activity towards *M*. *violaceum* at 2.3 μg mL^-1^ and moderate antibiotic activity. This study helps to link rare 1,3-benzodioxin-4-ones produced by *Syn*. *ericacearum* and other fungi within the Xylariales.

## Materials and methods

### General experimental section

NMR spectra of purified metabolites were recorded with a Bruker Avance 400 Spectrometer (Milton, Ontario) at 400.1 (^1^H) and 100 MHz (^13^C) using a 5 mm auto-tuning broadband probe with a Z-gradient. Secondary metabolites were dissolved in CD_3_OD (CDN Isotopes, Point Claire, Quebec) and referenced to the solvent peak (*δ*_H_ 3.31 and *δ*_C_ 49.1). LC-HRMS spectra were acquired with a Thermo Q-Exactive Orbitrap mass spectrometer (Thermo Scientific, Waltham, MA), coupled to an Agilent 1290 HPLC system. Extracts and metabolites were separated by an Agilent Zorbax Eclipse Plus, RRHD C_18_ (2.1 x 50 mm, 1.8 μm) column and a mobile phase consisting of acetonitrile (ACN) -ddH_2_O with 0.1% formic acid at a flow rate of 0.3 mL min^-1^. Normal phase and semi-preparative fractions were screened by LC-UV-MS with a Waters 2795 separation module, Waters 996 diode array detector, and Micromass Quatro LC mass spectrometer. Fractions were separated by a Phenomenex Kinetix C_18_ (100 × 4.60 mm, 2.6 μm) column (Torrance, CA, USA) using a mobile phase also consisting of ACN-ddH_2_O with 0.1% formic acid at a flow rate of 1.0 mL min^-1^. Semi-preparative HPLC was achieved with an Agilent 1100 HPLC system equipped with a diode array detector, Phenomnex Luna C_18_ (250 x 10.00 mm, 5 μm) column, and a mobile phase consisting of ACN-ddH_2_O. Gradients were programmed accordingly for each purified compounds with a flow rate of 4 mL min^-1^. Silica gel (Silicycle; 40–63 μm) was utilized for normal phase flash chromatography. Optical rotations were acquired with an Autopol IV polarimeter (Rudolph Analytical, Hackettown) and UV spectra were recorded with a Varian Cary 3 UV-VIS spectrophotometer scanning from 190–800 nm.

### Isolation and identification of fungal strains

Two collections of symptomatic leaves of *Kalmia latifolia* and *Rhododendron groenlandicum* were made during an ongoing survey of endophytes and other plant-associated fungi in Eastern Canada. Conidia from acervuli associated with leaf spots were transferred to Petri dishes containing 2% malt extract agar (MEA; 20 g Bacto malt extract, Difco Laboratories, Sparks, Maryland; 15 g agar, EMD Chemicals Inc., New Jersey; 1 L distilled water) and incubated at 20 °C. To induce sporulation, DAOMC 250336 was also grown on cornmeal agar (CMA; Acumedia Manufacturers Inc., Lansing, MI), oatmeal agar [49], and water agar (1.5% water agar (WA, with 1 mL trace metal solution; [50]) under 12:12 h fluorescent light and ambient natural light conditions. Additionally, MEA blocks (ca. 1 cm^3^) containing mycelia were placed in 9 cm Petri dishes containing ca. 25 μL sterile autoclaved tap water and exposed to ambient light.

Total genomic DNA was extracted from MEA cultures using the Ultraclean Microbial DNA isolation kit (Mo Bio Laboratories, Carlsbad, CA, USA) following the manufacturer’s protocol. The ITS region was amplified and sequenced using the primer pair V9G and LS266 [51,52]. Partial LSU was amplified using the primer pair LROR and LR5 and sequenced using LROR, LR3, LR3R, and LR5 [53]. PCR and sequencing protocol were carried out according to McMullin et al. [33]. Sequence contigs were assembled and trimmed using Geneious R8 v. 8.1.5 (Biomatters, Auckland, New Zealand).

ITS and LSU sequences of related Sporocadaceae species were obtained from GenBank by conducting BLAST searches using DAOMC 250336 and DAOMC 251621 as query sequences. A concatenated ITS and LSU dataset containing 59 sequences was aligned using MAFFT v. 7 [54] and visually examined in Geneious. MrModeltest v. 2.2.6 was used to determine the most suitable sequence evolution model (GTR+I+G) using the Akaike information criterion [55]. The ex-type culture of *Phlogicylindrium uniforme* (CBS 131312; Phlogicylindriaceae) was selected as outgroup because of its phylogenetic position outside of Sporocadaceae [19]. MrBayes v. 3.2 was used to perform Bayesian inference (BI) phylogenetic reconstruction [56]. Three independent Metropolis-coupled Markov chain Monte Carlo (MCMCMC) samplings were performed with 11 heated chains and one cold chain. Sampling occurred every 500 generations until the standard deviation of split frequencies reached a value < 0.01 (commands: stoprule = yes stopval = 0.01). The first 25% of trees were discarded as burn-in and the remaining trees were retained and combined into one 50% majority rule consensus tree. A maximum likelihood (ML) analysis was performed using RAxML v. 8.2.4 [57] in PAUP* v. 4.0b10 [58] starting from a random starting tree with 1000 bootstrap replicates. Trees were visualized in FigureTree 1.4.2 (available at http://tree.bio.ed.ac.uk/software/Figuretree/) and exported as SVG vector graphics for assembly in Adobe Illustrator v10 (Adobe Systems, San Jose, CA). Novel sequences used in this study were accessioned in GenBank and the novel species and associated metadata were deposited in MycoBank (www.mycobank.org). Collecting on Campobello Island was part of the 2016 Campobello Island Mycological Foray hosted by the New Brunswick Museum and J.D. Irving, Limited gave permission to conduct fungal collections on their sites.

### Nomenclature

The electronic version of this article in Portable Document Format (PDF) in a work with an ISSN or ISBN will represent a published work according to the International Code of Nomenclature for algae, fungi, and plants, and hence the new names contained in the electronic publication of a PLOS ONE article are effectively published under that Code from the electronic edition alone, so there is no longer any need to provide printed copies. In addition, new names contained in this work have been submitted to MycoBank from where they will be made available to the Global Names Index. The unique MycoBank number can be resolved and the associated information viewed through any standard web browser by appending the MycoBank number contained in this publication to the prefix http://www.mycobank.org/MB/. The online version of this work is archived and available from the following digital repositories: PubMed Central, LOCKSS.

### Fermentation and extraction

For inoculation into culture medium, *Syn*. *ericacearum* DAOMC 250336 growing on a 2% malt extract agar (MEA; Difco Laboratories) was excised and macerated in sterile ddH_2_O under aseptic conditions. Aliquots were used to inoculate (5% v/v) fifteen 250 mL Erlenmeyer flasks each containing 50 mL of 2% malt extract (Difco Laboratories) broth. First stage cultures were incubated for one week on a rotary shaker (100 RPM) in the dark at 25 °C. Resulting mycelia were macerated under aseptic conditions, and individually transferred to Glaxo bottles containing 1 L of the same culture medium. Second stage cultures were incubated stationary as described above for eight weeks.

After the incubation period, *Syn*. *ericacearum* mycelia were separated from the culture filtrate by suction through Whatman #4 (Whatman GE Healthcare, UK) filter papers. Mycelia were lyophilized and stored at -20 °C. The culture filtrate was saturated with NaCl and extracted with ACS grade EtOAc. The aqueous culture filtrate was discarded and the organic layer was filtered through anhydrous Na_2_SO_4_ and Whatman #1 filter papers prior to drying by rotary evaporation. The resulting culture filtrate extracts were dissolved in HPLC grade MeOH, passed through 0.2μm PTFE (25 mm) syringe filters (Tisch Scientific, USA), dried under a gentle stream of nitrogen gas and weighed. The resulting culture filtrate extract (1.20 g) was stored dry in an amber vial in a freezer at -20 °C.

### Metabolite isolation

The culture filtrate extract of *Syn*. *ericacearum* DAOMC 250336 was assessed for *in vitro* antifungal activity with a modified Oxford disc assay and subsequently chromatographed using a bioassay-guided approach. Flash column chromatography implementing a short Silica column and step gradient elution system of hexanes-EtOAc (0–100% v/v) in 10 percent increments followed by 5%, 10%, 25% and 50% EtOAc-MeOH (v/v) yielded 14 fractions screened for antifungal activity. Fractions 5–8, and 12–14 all exhibited antifungal activity with the former group showing a greater inhibitory effect *in vitro*. Based on LC-UV-MS, fractions with similar LC-UV-MS spectra were combined and metabolites were purified by reverse phase semi-preparative HPLC. Fractions 6–7 which eluted with 60–70% hexanes-EtOAc (v/v) were combined (48 mg) and further separated by semi-preparative HPLC with a linear ACN-ddH_2_O gradient programmed from 5–100% ACN over 25 minutes to afford compound **1** (14.9 mg). Compound **3** (46.0 mg) and additional compound **1** used for chemical derivatization were purified using the same semi-preparative HPLC method from fraction 8 (149.0 mg) that eluted with 80% hexanes-EtOAc. Fraction 12 (39.0 mg) eluted with 10% MeOH-EtOAc and provided compound **2** (6.9 mg) prior to further purification with a linear semi-preparative HPLC method programmed from 5–100% ACN over 20 minutes. ^1^H, ^13^C and HMBC spectra for natural products (**1**–**3**) can be found in the supporting information (Figures A-I in S1 File).

Synnemadoxin A (**1**): 14.9 mg; dull brown oil; [α]^23^_D_ 9.4 (c 0.3, MeOH), [α]^23^_D_ 9.8 (c 0.3, CHCl_3_); UV (MeOH) λ_max_ (log ε) 224 (4.23), 268 (3.84), 306 (3.23); for ^1^H and ^13^C NMR spectroscopic data, see Table 1; HRMS *m/z* 293.1033 [M-H]^-^ (calc. for [C_15_H_17_O_6_]^-^ 293.1031).
Synnemadoxin B (**2**): 6.9 mg; dull brown oil; [α]^23^_D_ 10.4 (c 0.3, MeOH), [α]^23^_D_ 10.2 (c 0.3, CHCl_3_); UV (MeOH) λ_max_ (log ε) 224 (4.34), 268 (3.58), 306 (3.21); for ^1^H and ^13^C NMR spectroscopic data, see Table 1; HRMS *m/z* 309.0981 [M-H]^-^ (calc. for [C_15_H_17_O_7_]^-^ 309.0980).
Synnemadiacid A (**3**): 46.0 mg; yellow oil; UV (MeOH) λ_max_ (log ε) 221 (4.12), 270 (3.53), 310 (3.14); for ^1^H and ^13^C NMR spectroscopic data, see Table 2; HRMS *m/z* 293.1032 [M-H]^-^ (calc. for [C_15_H_17_O_6_]^-^ 293.1031).

### *In silico* computed chemical shifts

Theoretical chemical shifts were calculated for the four possible stereoisomers of compounds **1**, and **1a** based on a computational method described by Willoughby et al. [17], with some minor modifications. Briefly, the AMBER12 program suite [59] using the Generalized AMBER Force Field (GAFF) was used to explore the conformational space of each stereoisomer [60]. Minimized structures were heated to 500K over 5 ps (1 fs time step) and equilibrated at 500K for 1000, 100 ps cycles (0.5 fs time step). Following each cycle, structures were cooled to 0K over 5 ps (1 fs time step). Unique conformations were identified manually based on the C-2-Me, C-2, C-9, C-9-Me dihedral angles, and potential energy recorded following each cycle. Up to 20 conformations for each stereoisomer were removed for geometric optimization and frequency calculations using the B3LYP functional with a 6–31+G(d,p) basis set [61]. NMR shielding tensors were subsequently calculated from the geometry optimized structures with the B3LYP functional with the 6-311G (2d,p) basis set. Duplicate conformations with identical shielding tensors and zero point energies were removed and the chemical shift contribution for each conformation were averaged based on Boltzmann distributions. The scaling factor and intercept were determined using a linear fit of the theoretical tensors of all protons not predicted to be affected by the stereocenters of interest (positions 8, 5-Me, 6-Me and 7-OMe). The scaling factor and intercept were -0.99972 and 31.8085, respectively.

### Antimicrobial assays

Initial antifungal activity of the *Syn*. *ericacearum* DAOMC 250336 culture filtrate extract (50 mg mL^-1^) and flash chromatography fractions were screened using a modified Oxford diffusion assay against *Microbotryum violaceum* and *Saccharomyces cerevisiae* homogeneously spread on 2% MEA as previously described by McMullin et al. [33]. Purified natural products **1–3** were tested for *in vitro* antimicrobial activity against *M*. *violaceum*, *S*. *cerevisiae*, *Bacillus subtilis* (ATCC 23857) and *Escherichia coli* (ATCC 67878). *M*. *violaceum* was grown in 20 g L^-1^ malt extract (Bacto), 2.5 g L^-1^ peptone (Bacto) and 2.5 g L^-1^ yeast extract (Sigma, St. Louis, MO, U.S.A.) whereas *S*. *cerevisiae* was inoculated and grown in 1 g L^-1^ yeast extract supplemented with 10 g L^-1^ glucose. Bacteria were inoculated and grown in 5 g L^-1^ yeast extract, 10 g L^-1^ peptone, and 10 g L^-1^ NaCl. Nystatin was the positive control for antifungal assays and chloramphenicol was the antibacterial positive control. DMSO was the negative control for all *in vitro* assays. Isolated metabolites and positive controls were individually tested at 150, 75, 37.5, 18.8, 9.3, 4.7, 2.3 and 1.2 μg mL^-1^ in sterile 96-well microplates, respectively (Falcon 353072 Microtest-9, Franklin Lakes, NJ, U.S.A.). A 10 μL aliquot of each individual metabolite solution dissolved in DMSO was added to 200 μL fungal or bacterial suspension. Assays were performed in triplicate and incubated at 25 °C with a rotary table shaker providing gentle agitation (500 rpm). Optical density (OD) measurements were made at 600 nm with a Molecular Devices Spectra Max 340PC reader (Sunnyvale, CA, U.S.A.). Antimicrobial OD data were subsequently analyzed by ANOVA followed by Tukey’s test (*p* < 0.05) for significant differences (Systat V13.1; Systat Software Inc., Chicago, IL, U.S.A.) compared to the negative control DMSO. Positive controls inhibited the growth of all test organisms at the second lowest concentrations tested. Previous studies had shown the MIC of nystatin in the *S*. *cerevisiae* culture used was 4 μM and for *M*. *violaceum*, 2 μM. In the antibiotic assays, for *B*. *subtilis* chloramphenicol had an MIC of 2.5 μM. All test organisms grew in the presence of the negative control DMSO.

## Supporting information

S1 File Supporting Information: Figure A. ^1^H (400 MHz, CD_3_OD) NMR spectrum for **1**. Figure B. ^13^C (100 MHz, CD_3_OD) NMR spectrum for **1**. Figure C. HMBC NMR spectrum for **1** in CD_3_OD. Figure D. ^1^H (400 MHz, CD_3_OD) NMR spectrum for **2**. Figure E. ^13^C (100 MHz, CD_3_OD) NMR spectrum for **2**. Figure F. HMBC NMR spectrum for **2** in CD_3_OD. Figure G. ^1^H (400 MHz, CD_3_OD) NMR spectrum for **3**. Figure H. ^13^C (100 MHz, CD_3_OD) NMR spectrum for **3**. Figure I. HMBC NMR spectrum for **3** in CD_3_OD. Table A. Comparison of experimental and computed ^1^H chemical shift data (ppm) for **1**. Table B. Comparison of experimental and computed ^13^C chemical shift data (ppm) for **1**. Table C. Comparison of experimental and computed ^1^H chemical shift data (ppm) for **1a**. Table D. Comparison of experimental and computed ^13^C chemical shift data (ppm) for **1a**. Figure J. Methylation of synnemadoxin A (**1**) and synnemadiacid A (**3**) to their respective hexamethyl (**1a**) and heptamethyl (**3a**) derivatives using excess diazomethane. Table E. 1H (400 MHz) and 13C (100 MHz) NMR data for hexamethyl derivative of **1** (**1a**) in CD3OD. Table F. 1H (400 MHz) and 13C (100 MHz) NMR data for heptamethyl derivative of **3** (**3a**) in CD3OD. Text A. Methylation of synnemadoxin A (1) and synnemadiacid A (3) to their respective hexamethyl (1a) and heptamethyl (3a) derivatives using excess diazomethane.
(DOCX)
