# Supplementary material for: New 1,3-benzodioxin-4-ones from Synnemapestaloides ericacearum sp. nov., a biosynthetic link to remarkable compounds within the Xylariales
Source: PLoS One. 2018 Jun 27;13(6):e0198321. doi: 10.1371/journal.pone.0198321 (PMC6021072; doi:10.1371/journal.pone.0198321)
Supplement: S1 File — Supporting Information: Figure A. 1H (400 MHz, CD3OD) NMR spectrum for 1. Figure B. 13C (100 MHz, CD3OD) NMR spectrum for 1. Figure C. HMBC NMR spectrum for 1 in CD3OD. Figure D. 1H (400 MHz, CD3OD) NMR spectrum for 2. Figure E. 13C (100 MHz, CD3OD) NMR spectrum for 2. Figure F. HMBC NMR spectrum for 2 in CD3OD. Figure G. 1H (400 MHz, CD3OD) NMR spectrum for 3. Figure H. 13C (100 MHz, CD3OD) NMR spectrum for 3. Figure I. HMBC NMR spectrum for 3 in CD3OD. Table A. Comparison of experimental and computed 1H chemical shift data (ppm) for 1. Table B. Comparison of experimental and computed 13C chemical shift data (ppm) for 1. Table C. Comparison of experimental and computed 1H chemical shift data (ppm) for 1a. Table D. Comparison of experimental and computed 13C chemical shift data (ppm) for 1a. Figure J. Methylation of synnemadoxin A (1) and synnemadiacid A (3) to their respective hexamethyl (1a) and heptamethyl (3a) derivatives using excess diazomethane. Table E. 1H (400 MHz) and 13C (100 MHz) NMR data for hexamethyl derivative of 1 (1a) in CD3OD. Table F. 1H (400 MHz) and 13C (100 MHz) NMR data for heptamethyl derivative of 3 (3a) in CD3OD. Text A. Methylation of synnemadoxin A (1) and synnemadiacid A (3) to their respective hexamethyl (1a) and heptamethyl (3a) derivatives using excess diazomethane. (DOCX) [file pone.0198321.s001.docx]

**Supporting Information**

Figure A. ^1^H (400 MHz, CD_3_OD) NMR spectrum for **1**.

Figure B. ^13^C (100 MHz, CD_3_OD) NMR spectrum for **1**.

Figure C. HMBC NMR spectrum for **1** in CD_3_OD.

Figure D. ^1^H (400 MHz, CD_3_OD) NMR spectrum for **2**.

Figure E. ^13^C (100 MHz, CD_3_OD) NMR spectrum for **2**.

Figure F. HMBC NMR spectrum for **2** in CD_3_OD.

Figure G. ^1^H (400 MHz, CD_3_OD) NMR spectrum for **3**.

Figure H. ^13^C (100 MHz, CD_3_OD) NMR spectrum for **3**.

Figure I. HMBC NMR spectrum for **3** in CD_3_OD.

Table A. Comparison of experimental and computed ^1^H chemical shift data (ppm) for **1**.

Table B. Comparison of experimental and computed ^13^C chemical shift data (ppm) for **1**.

Table C. Comparison of experimental and computed ^1^H chemical shift data (ppm) for **1a**.

Table D. Comparison of experimental and computed ^13^C chemical shift data (ppm) for **1a**.

Figure J. Methylation of synnemadoxin A (**1**) and synnemadiacid A (**3**) to their respective hexamethyl (**1a**) and heptamethyl (**3a**) derivatives using excess diazomethane.

Table E. ^1^H (400 MHz) and ^13^C (100 MHz) NMR data for hexamethyl derivative of **1** (**1a**) in CD_3_OD.

Table F. ^1^H (400 MHz) and ^13^C (100 MHz) NMR data for heptamethyl derivative of **3** (**3a**) in CD_3_OD.

Text A. Methylation of synnemadoxin A (**1**) and synnemadiacid A (**3**) to their respective hexamethyl (**1a**) and heptamethyl (**3a**) derivatives using excess diazomethane.

Figure A. ^1^H (400 MHz, CD_3_OD) NMR spectrum for **1**.




Figure B. ^13^C (100 MHz, CD_3_OD) NMR spectrum for **1**.

Figure C. HMBC NMR spectrum for **1** in CD_3_OD.

Figure D. ^1^H (400 MHz, CD_3_OD) NMR spectrum for **2**.




Figure E. ^13^C (100 MHz, CD_3_OD) NMR spectrum for **2**.

Figure F. HMBC NMR spectrum for **2** in CD_3_OD.

Figure G. ^1^H (400 MHz, CD_3_OD) NMR spectrum for **3**.




Figure H. ^13^C (100 MHz, CD_3_OD) NMR spectrum for **3**.

Figure I. HMBC NMR spectrum for **3** in CD_3_OD.

Table A. Comparison of experimental and computed ^1^H chemical shift data (ppm) for **1**.

|  |  | Computed ^1^H chemical shifts (ppm) for **1** | | | | Absolute chemical shift error (ppm) | | | |
| --- | --- | --- | --- | --- | --- | --- | --- | --- | --- |
| Position | *δ*_H_ exp | 2*R*, 9*R* | 2*R*, 9*S* | 2*S*, 9*R* | 2*S*, 9*S* | 2*R*, 9*R* | 2*R*, 9*S* | 2*S*, 9*R* | 2*S*, 9*S* |
| 8 | 6.51 | 6.5025 | 6.5348 | 6.5410 | 6.4714 | 0.0075 | 0.0248 | 0.0310 | 0.0386 |
| 5-Me | 2.58 | 2.5244 | 2.5243 | 2.5391 | 2.5364 | 0.0556 | 0.0557 | 0.0409 | 0.0436 |
| 6-Me | 2.11 | 2.1472 | 2.1433 | 2.1565 | 2.1589 | 0.0372 | 0.0333 | 0.0465 | 0.0489 |
| 7-OMe | 3.87 | 3.9166 | 3.9298 | 3.9377 | 3.9055 | 0.0366 | 0.0498 | 0.0577 | 0.0255 |
| 2-Me | 1.73 | 1.6631 | 1.6065 | 1.6785 | 1.7359 | 0.0669 | 0.1235 | 0.0515 | 0.0059 |
| 9 | 3.18 | 3.2696 | 3.1595 | 3.4799 | 3.6258 | 0.0896 | 0.0205 | 0.2999 | 0.4458 |
| 9-Me | 1.28 | 1.3803 | 1.3679 | 1.2858 | 1.3168 | 0.1003 | 0.0879 | 0.0058 | 0.0368 |
|  |  |  |  | MAE (ppm) | | 0.0562 | 0.0565 | 0.0762 | 0.0922 |

Table B. Comparison of experimental and computed ^13^C chemical shift data (ppm) for **1**.

|  |  | Computed ^13^C chemical shifts (ppm) for **1** | | | | Absolute chemical shift error (ppm) | | | |
| --- | --- | --- | --- | --- | --- | --- | --- | --- | --- |
| Position | *δ*_C_ exp | 2*R*, 9*R* | 2*R*, 9*S* | 2*S*, 9*R* | 2*S*, 9*S* | 2*R*, 9*R* | 2*R*, 9*S* | 2*S*, 9*R* | 2*S*, 9*S* |
| 5 | 143.2 | 146.5354 | 146.4258 | 146.5469 | 146.7018 | 3.3354 | 3.2258 | 3.3469 | 3.5018 |
| 8 | 98.1 | 95.4766 | 95.4191 | 95.6515 | 95.6994 | 2.5834 | 2.6409 | 2.4085 | 2.3606 |
| 7 | 165.0 | 163.7772 | 163.7199 | 163.7790 | 164.1232 | 1.1928 | 1.2501 | 1.1910 | 0.8468 |
| 6 | 122.7 | 122.8881 | 122.9298 | 123.0776 | 122.8883 | 0.1881 | 0.2298 | 0.3776 | 0.1883 |
| 5-Me | 17.4 | 19.0650 | 19.0313 | 19.0634 | 19.1427 | 1.6750 | 1.6413 | 1.6734 | 1.7527 |
| 6-Me | 11.3 | 11.3569 | 11.3617 | 11.3826 | 11.4168 | 0.0569 | 0.0617 | 0.0826 | 0.1168 |
| 7-OMe | 56.6 | 55.1236 | 55.1341 | 55.1619 | 55.1073 | 1.4964 | 1.4859 | 1.4581 | 1.5127 |
| 2 | 107.2 | 108.2859 | 107.3357 | 108.8998 | 109.1472 | 1.0959 | 0.1457 | 1.7098 | 1.9572 |
| 2-Me | 20.9 | 18.1747 | 20.6232 | 19.6707 | 19.8912 | 2.7453 | 0.2968 | 1.2493 | 1.0288 |
| 9 | 47.8 | 52.0698 | 51.2473 | 48.1673 | 47.6105 | 4.2498 | 3.4273 | 0.3473 | 0.2095 |
| 10 | 175.4 | 175.4068 | 175.1577 | 175.6382 | 175.5375 | 0.0468 | 0.2023 | 0.2782 | 0.1775 |
| 9-Me | 12.9 | 13.3559 | 12.4802 | 13.7148 | 14.2732 | 0.5059 | 0.3698 | 0.8648 | 1.4232 |
| 4 | 161.9 | 161.2172 | 161.5437 | 161.2919 | 161.1095 | 0.7028 | 0.3763 | 0.6281 | 0.8105 |
| 4a | 106.2 | 104.9962 | 104.9978 | 105.1247 | 104.6347 | 1.1638 | 1.1622 | 1.0353 | 1.5253 |
| 8a | 157.7 | 157.1952 | 157.1951 | 156.8928 | 157.2864 | 0.4548 | 0.4549 | 0.7572 | 0.3636 |
|  |  |  |  | MAE (ppm) | | 1.3707 | 0.8044 | 0.8587 | 0.9370 |

Table C. Comparison of experimental and computed ^1^H chemical shift data (ppm) for **1a**.

|  |  | Computed ^1^H chemical shifts (ppm) for **1a** | | | | Absolute chemical shift error (ppm) | | | |
| --- | --- | --- | --- | --- | --- | --- | --- | --- | --- |
| Position | *δ*_H_ exp | 2*R*, 9*R* | 2*R*, 9*S* | 2*S*, 9*R* | 2*S*, 9*S* | 2*R*, 9*R* | 2*R*, 9*S* | 2*S*, 9*R* | 2*S*, 9*S* |
| 8 | 6.51 | 6.4869 | 6.5190 | 6.5219 | 6.4620 | 0.0231 | 0.0090 | 0.0119 | 0.0480 |
| 5-Me | 2.59 | 2.5188 | 2.5277 | 2.5316 | 2.5256 | 0.0712 | 0.0623 | 0.0584 | 0.0644 |
| 6-Me | 2.12 | 2.1472 | 2.1401 | 2.1411 | 2.1502 | 0.0272 | 0.0201 | 0.0211 | 0.0302 |
| 7-OMe | 3.88 | 3.9149 | 3.9356 | 3.9289 | 3.9057 | 0.0349 | 0.0556 | 0.0489 | 0.0257 |
| 2-Me | 1.70 | 1.6718 | 1.5360 | 1.6173 | 1.7094 | 0.0282 | 0.1640 | 0.0827 | 0.0094 |
| 9 | 3.27 | 3.3471 | 3.0724 | 3.2437 | 3.4485 | 0.0771 | 0.1976 | 0.0263 | 0.1785 |
| 9-Me | 1.27 | 1.3023 | 1.3282 | 1.2939 | 1.2779 | 0.0323 | 0.0582 | 0.0239 | 0.0079 |
| 10-OMe | 3.71 | 3.7401 | 3.7859 | 3.7826 | 3.7314 | 0.0301 | 0.0759 | 0.0726 | 0.0214 |
|  |  |  |  | MAE (ppm) | | 0.0419 | 0.1239 | 0.0514 | 0.0543 |

Table D. Comparison of experimental and computed ^13^C chemical shift data (ppm) for **1a**.

|  |  | Computed ^13^C chemical shifts (ppm) for **1a** | | | | Absolute chemical shift error (ppm) | | | |
| --- | --- | --- | --- | --- | --- | --- | --- | --- | --- |
| Position | *δ*_C_ exp | 2*R*, 9*R* | 2*R*, 9*S* | 2*S*, 9*R* | 2*S*, 9*S* | 2*R*, 9*R* | 2*R*, 9*S* | 2*S*, 9*R* | 2*S*, 9*S* |
| 5 | 143.3 | 146.4937 | 146.2894 | 146.3155 | 146.5066 | 3.1937 | 2.9894 | 3.0155 | 3.2066 |
| 8 | 106.0 | 104.8162 | 105.2582 | 105.3068 | 104.8649 | 1.1838 | 0.7418 | 0.6932 | 1.1351 |
| 7 | 157.5 | 157.6407 | 157.0714 | 156.9516 | 157.6039 | 0.1407 | 0.4286 | 0.5484 | 0.1039 |
| 6 | 98.1 | 95.6587 | 95.2188 | 95.4376 | 95.6485 | 2.4413 | 2.8812 | 2.6624 | 2.4515 |
| 5-Me | 165.1 | 163.9456 | 163.6495 | 163.6564 | 163.9869 | 1.1544 | 1.4505 | 1.4436 | 1.1131 |
| 6-Me | 122.9 | 122.7348 | 122.7718 | 122.8462 | 122.7431 | 0.1652 | 0.1282 | 0.0538 | 0.1569 |
| 7-OMe | 17.4 | 19.1268 | 19.0217 | 19.0247 | 19.1586 | 1.7268 | 1.6217 | 1.6247 | 1.7586 |
| 2 | 11.3 | 11.3566 | 11.3422 | 11.3335 | 11.3792 | 0.0566 | 0.0422 | 0.0335 | 0.0792 |
| 2-Me | 56.5 | 55.1052 | 55.1582 | 55.1449 | 55.0899 | 1.3948 | 1.3418 | 1.3551 | 1.4101 |
| 9 | 107.0 | 109.2674 | 107.4558 | 108.4867 | 109.4667 | 2.2674 | 0.4558 | 1.4867 | 2.4667 |
| 10 | 20.9 | 18.6987 | 20.8541 | 19.4871 | 18.3962 | 2.2013 | 0.0459 | 1.4129 | 2.5038 |
| 9-Me | 47.2 | 50.8426 | 52.4919 | 51.2656 | 49.9908 | 3.6426 | 5.2919 | 4.0656 | 2.7908 |
| 4 | 173.6 | 176.3972 | 175.8160 | 176.1619 | 176.5427 | 2.7972 | 2.2160 | 2.5619 | 2.9427 |
| 4a | 12.6 | 13.9459 | 12.0931 | 13.0409 | 14.1672 | 1.3459 | 0.5069 | 0.4409 | 1.5672 |
| 8a | 161.7 | 161.3258 | 161.6859 | 161.5173 | 161.3116 | 0.3742 | 0.0141 | 0.1827 | 0.3884 |
| 10-OMe | 52.5 | 53.1527 | 53.1804 | 53.2593 | 53.1867 | 0.6527 | 0.6804 | 0.7593 | 0.6867 |
|  |  |  |  | MAE (ppm) | | 1.8973 | 1.3159 | 1.5586 | 1.9066 |

Figure J. Methylation of synnemadoxin A (**1**) and synnemadiacid A (**3**) to their respective hexamethyl (**1a**) and heptamethyl (**3a**) derivatives using excess diazomethane.





Table E. ^1^H (400 MHz) and ^13^C (100 MHz) NMR data for hexamethyl derivative of **1** (**1a**) in CD_3_OD.

|  |  | | **1a** | |  | |
| --- | --- | --- | --- | --- | --- | --- |
| **Position** |  |  | ***δ*_C,_ type** | ***δ*_H_ (*J* in Hz)** |  |  |
| **2** |  |  | 107.0, C |  |  |  |
| **4** |  |  | 161.7, C |  |  |  |
| **4a** |  |  | 106.0, C |  |  |  |
| **5** |  |  | 143.3, C |  |  |  |
| **6** |  |  | 122.9, C |  |  |  |
| **7** |  |  | 165.1, C |  |  |  |
| **8** |  |  | 98.1, CH | 6.51, s |  |  |
| **8a** |  |  | 157.5, C |  |  |  |
| **9** |  |  | 47.2, CH | 3.27, m |  |  |
| **10** |  |  | 173.6, C |  |  |  |
| **2-Me** |  |  | 20.9, CH_3_ | 1.70, s |  |  |
| **5-Me** |  |  | 17.4, CH_3_ | 2.59, s |  |  |
| **6-Me** |  |  | 11.3, CH_3_ | 2.12, s |  |  |
| **9-Me** |  |  | 12.6, CH_3_ | 1.27, d (7.0) |  |  |
| **7-OMe** |  |  | 56.5, CH_3_ | 3.88, s |  |  |
| **10-OMe** |  |  | 52.5, CH_3_ | 3.71, s |  |  |

Table F. ^1^H (400 MHz) and ^13^C (100 MHz) NMR data for heptamethyl derivative of **3** (**3a**) in CD_3_OD.

|  |  | | **3a** | |
| --- | --- | --- | --- | --- |
| **Position** |  |  | **δ_C,_ type** | ***δ*_H_ (*J* in Hz)** |
| **1** |  |  | 120.9, C |  |
| **2** |  |  | 137.0, C |  |
| **3** |  |  | 122.6, C |  |
| **4** |  |  | 160.4, C |  |
| **5** |  |  | 101.2, CH | 6.39, s |
| **6** |  |  | 151.4, C |  |
| **2-Me** |  |  | 17.0, CH_3_ | 2.20, s |
| **3-Me** |  |  | 11.5, CH_3_ | 2.12, s |
| **1’** |  |  | 163.1, C |  |
| **2’** |  |  | 112.3, C |  |
| **3’** |  |  | 170.8, C |  |
| **1’-Me** |  |  | 17.4, CH_3_ | 2.17, q (1.4) |
| **2’-Me** |  |  | 12.3, CH_3_ | 1.89, q (1.4) |
| **1”** |  |  | 170.1, C |  |
| **4-OMe** |  |  | 56.3, CH_3_ | 3.79, s |
| **3’-OMe** |  |  | 51.9, CH_3_ | 3.74, s |
| **1”-OMe** |  |  | 52.6, CH_3_ | 3.81, s |

Text A. Methylation of synnemadoxin A (1) and synnemadiacid A (3) to their respective hexamethyl (1a) and heptamethyl (3a) derivatives using excess diazomethane.

Methylation of **1** with excess diazomethane yielded a hexamethyl derivative (1a), identifying one exchangeable acidic proton within the structure (S14 Fig.). An HMBC correlation from the derivatized methoxy at *δ* 3.71 (10-OMe) to *δ* 173.6 (C-10) in 1a and the very similar chemical shifts for both the native natural product (**1**) and methylated derivative (**1a**) confirmed a carboxylic acid functionality at C-10 within the substituted 3,3-dioxy-2-methylbutanoic acid moiety of **1** (S15 Table). The hexyamethyl derivative (**1a**) also revealed the four remaining oxygen atoms in **1** are not primary or secondary alcohols because they were not methylated by diazomethane. Compound **3** was also treated with excess diazomethane which yielded its heptamethyl derivative (**3a**), indicative of two exchangeable acidic protons (S14 Fig). HMBC correlations were observed from the two synthetic methoxy functionalities at δ 3.81 (s) and 3.74 (s) to respective carbonyls at δ 170.1 and 170.8 showing **3** possessed two carboxylic acid groups instead of one as observed for synnemadoxin A and B (S6 Table).

Compounds **1** (6.0 mg) and **3** (6.0 mg) were each dissolved in 500 µL of HPLC grade MeOH, and excess diazomethane was added at room temperature. The reaction mixture was stirred until nitrogen gas liberation ceased and dried under a gentle stream of nitrogen gas. Methylated derivatives **1a** and **3a** were purified by semi-preparative HPLC as described for compounds **1** and **3** and subjected to NMR spectroscopic analysis (S15 and S16 Tables).
